# Clinical Trial Data Management in Environmental Health Tailored for an African Setting

**DOI:** 10.3390/ijerph17020402

**Published:** 2020-01-08

**Authors:** Patricia Nicole Albers, Caradee Yael Wright

**Affiliations:** 1 Department of Geography, Geoinformatics and Meteorology, University of Pretoria, Pretoria 0028, South Africa; cwright@mrc.ac.za; 2 Environment and Health Research Unit, South African Medical Research Council, Pretoria 0084, South Africa

**Keywords:** data, methods, clinical trial, environmental health, sun exposure, public health, Python, SQLite, South Africa

## Abstract

Clinical trial data management tools are widely available—some free to access and others relatively expensive, particularly for low- and middle-income countries. Such tools also do not always permit adaptation for local conditions nor include options to capture environmental and meteorological data. In the context of climate change and pressing environmental health threats, more studies that aim to assess the impacts of environmental change on public health are being carried out. Here, using freely available software, we tailor-made a clinical trial data management tool that managed all aspects of an intervention-based clinical trial to assess the impact of personal solar ultraviolet radiation exposure on vaccine effectiveness. Data captured and associated procedures included patient data, scheduling, reporting, analysis and data management. Moreover, patient enrolment, recruitment, follow-up and decision-making in response to patient data were managed. Given the multidisciplinary study approach, the tool also managed all environmental and meteorological data for the rural African study site. Application of the tool ensured efficient communication between rural sites, a relatively high overall participant response rate (87%) and minimal loss to follow-up. This study suggests that it is possible to tailor-make a clinical trial data management tool for environmental and public health studies.

## 1. Introduction

Good Clinical Practice (GCP) [1] calls for excellent data management in research, especially when conducting clinical trials. Data management includes collection, quality, recording, maintenance and retrieval of source data from a clinical trial. The aim of data management is to transform responses from the study participants, efficiently and without errors, into data that are accurate and accessible. From here, it can be transformed into information that can be disseminated and understood through statistical analysis. Data management needs to be ordered using a standard operating procedure that includes checklists for assessment at each stage of a research project. Good data management ensures data integrity and accuracy [1].

In health sciences, clinical trials are used to understand diseases as well as to test the safety and/or effectiveness of a drug, therapy or experimental treatment. Clinical trials are prospective studies used to establish a causal relationship between an exposure and an outcome. They can be classified as treatment, screening, quality of life, genetics and prevention trials [2,3]. All clinical trials must comply with GCP principles and ensure excellent data management [4]. To do so as efficiently and as cost-effectively as possible, researchers use clinical trial data management tools and/or software. Many different types of clinical data management systems and tools exist [5] for example, Clincapture (https://www.getapp.com/industries-software/a/clincapture/) and IBM Clinical Development (https://www.ibm.com/za-en/marketplace/clinical-development). However, to date, clinical trials have rarely been used in environmental health research with less than 1% of environmental health studies applying the randomized clinical trial study design [6]. Environmental health aims to assess and control all the environmental determinants of health and wellbeing with the goal to prevent adverse exposures and disease and to create health-supportive environments [7]. Some argue that clinical equipoise prohibits randomized clinical trials in environmental health where communities in the control arm are placed in environments with known adverse environmental risks and/or interventions that are unaffordable to them post-study [8]. Exposure-health relationships such as those in environmental health are likely best to consider acute or intermediate effects rather than study diseases with long latency periods and that expose people for long durations. Importantly, the randomized clinical trial design may be useful for testing the efficacy of interventions to reduce exposure, as was done in this study [9].

As part of a larger intervention and control study that aimed to investigate whether or not providing sun protection to mothers of children prost-vaccination would elicit a more effective immune response in the child [9], we developed a clinical trial data management tool to assist in the study execution. While open-source tools for clinical data management in remote settings do exist [8,10], none met all the requirements for our proposed study and all its elements. Hence, the tool was designed with two purposes in mind: (1) to try to overcome potentially difficult socio-economic and socio-cultural conditions at the study sites that were envisaged to make aspects of clinical trial management challenging to maintain; and (2) to accommodate environmental and meteorological data capture and management in the tool. At the time of study preparation, no software was available to assist with environmental and meteorological data management components in the clinical trial study.

Therefore, the aim of this work was to design, implement and evaluate a clinical trial data management tool for an environmental health study in a rural African setting. Here, we explain the background to the study, the software used in the tool design process, implementation of the data management tool and evaluate the successes and shortfalls of the tool in relation to the completed study. Our findings are useful for researchers embarking on environmental health clinical trial and intervention studies in rural, low- and middle-income countries or for researchers needing to manage timelines and separate data collection tools such as questionnaires, checklists, timing of interventions, blood collection as well as environmental and meteorological datasets.

## 2. Methods

### 2.1. Clinical Trial Methods in Brief

**Figure 1:**
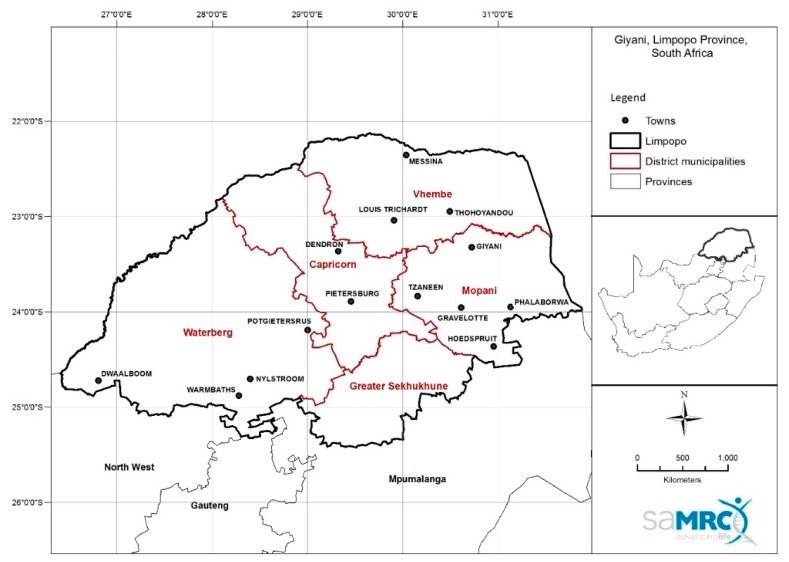
General location of the study clinics in Mopani District Municipality in the Limpopo Province of South Africa. The town of Giyani where the two study clinics were located is in the northern parts of the Mopani District Municipality (Map drawn by Thandi Kapwata of the South African Medical Research Council).

For a complete study protocol, refer to Wright et al. [9]. In brief, the study took place in two rural community health clinics in the Greater Giyani Local Municipality (Limpopo Province, South Africa) (Figure 1) during summer and autumn of 2015/2016 when ambient solar ultraviolet radiation levels are relatively high in the Southern Hemisphere. Two sites from the area were randomly selected and then randomized to the intervention and control arms.

**Figure 2:**
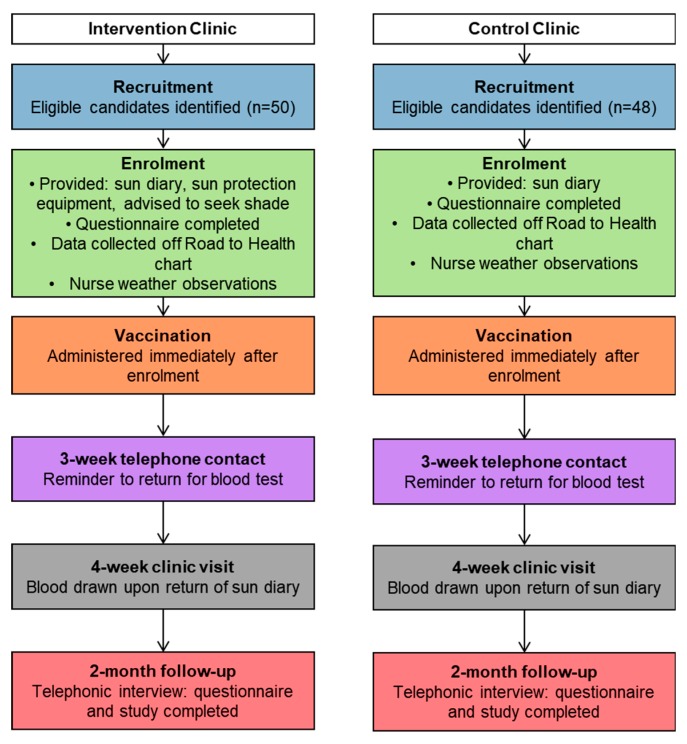
Participant flow through the study at the intervention and control clinics.

Eligible participants were recruited at each clinic consecutively. Participants were children aged 18 months or older and were reporting to either clinic for their booster (second) measles vaccination. The participant flow through the study is given in Figure 2. Children at control and intervention sites then received the measles vaccination intramuscularly according to the protocol of the South African National Health Department Extended Programme on Immunization [11] and using the government standard vaccine preparation method. Participants were asked to return to the clinic approximately four weeks later for blood testing. Unique identifier codes were allocated to each child participant and used to match all their data.

### 2.2. Ethics Approval and Consent to Participate

The protocol for recruitment, data and sample collection for the study was approved by the South African Medical Research Council Ethics Committee (EC013-4/2015). Informed consent was obtained from mothers of children who were enrolled in the study. The South African National Department of Health Clinical Trial Registration Number is DOH-27-1116-5591 (11 November 2016, retrospective registration). The Pan African Clinical Trial Registry Identification Number is PACTR201611001881114 (24 November 2016, retrospective registration). Research governance approval was provided through a peer-review process managed by the National Research Foundation of South Africa (Grant number 93426).

### 2.3. Data Management Tool Conceptulisation

The study design included several separate data collection tools and stages at which these were to be used, which differed slightly for the control and intervention groups. As a result, a data entry and study management tool was created. The nature of this specific research project informed the conceptualization of the tool. The stages and corresponding data collection sheets used in the study were: enrolment and an accompanying criteria checklist, collection of data from their Road to Health chart (date of first measles vaccination etc.), study questionnaire, a self-completed sun diary and a follow-up telephonic interview. An over-arching form detailing the management of all these stages was also included for the research nurses to complete. This form detailed the participants’ movement through the entire study process (Figure 2). There was also a nurse observation form to capture details about daily weather conditions, as well as on-site weather and solar ultraviolet radiation (UVR) data.

**Figure 3:**
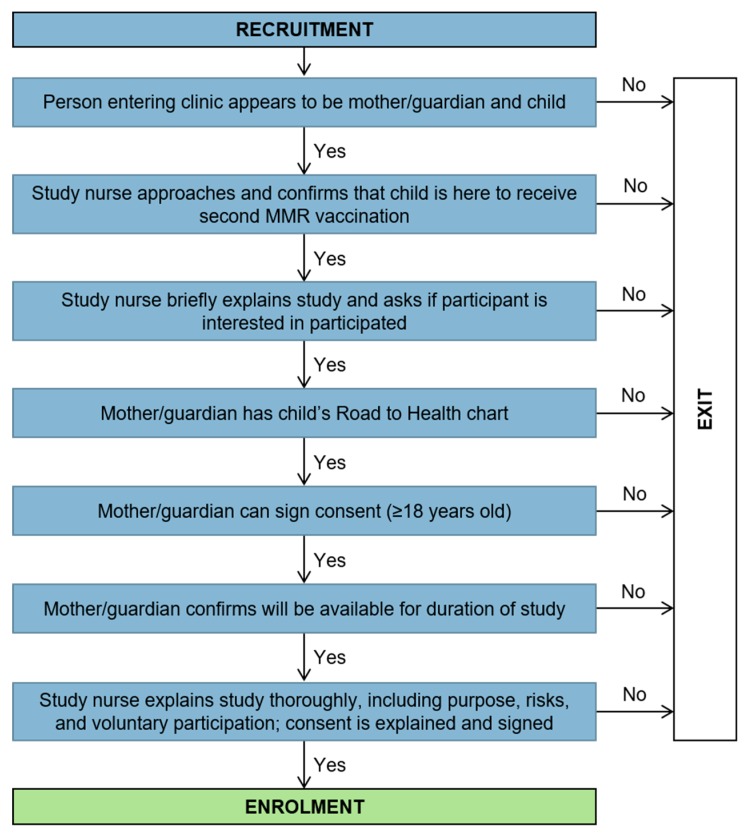
Recruitment stage flow chart.

**Figure 4:**
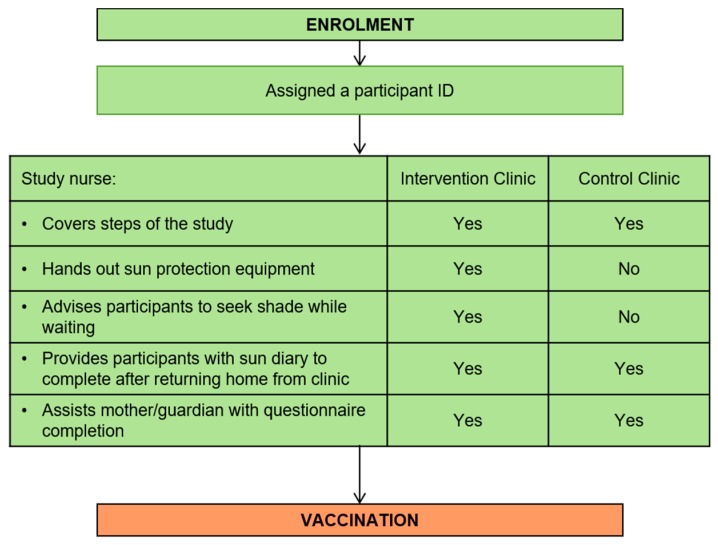
Enrolment stage flow chart.

All of the stages were pre-planned, managed and directed by the ‘flow chart of events’ and the accompanying ‘participant flow checklist’. The design of the tool was based on these stages and charts and transformed in an electronic version to assist the study team with managing and following the progress of the study given the remoteness of the study area. The tool matched the design of the study, where all the data collection forms in the study were recreated in an electronic database. It housed separate tables for each of these data collection tools as well as a form for the participant flow (see Figure 2). Following the recruitment stage (Figure 3) the participant then moved to the enrolment stage (Figure 4) where they were assigned a unique identifier and the consent form was signed. During this stage, several data forms were completed, namely an enrolment checklist, data from the child’s Road to Health chart, a questionnaire and nurse observations of daily weather, and a participant flow checklist was initiated.

**Figure 5:**
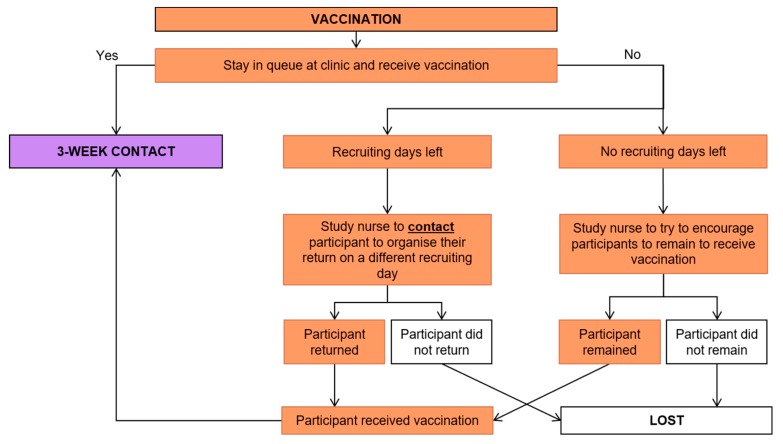
Vaccination stage flow chart.

Child participants then received the vaccination according to the National Department of Health guidelines [11] (Figure 5).

**Figure 6:**
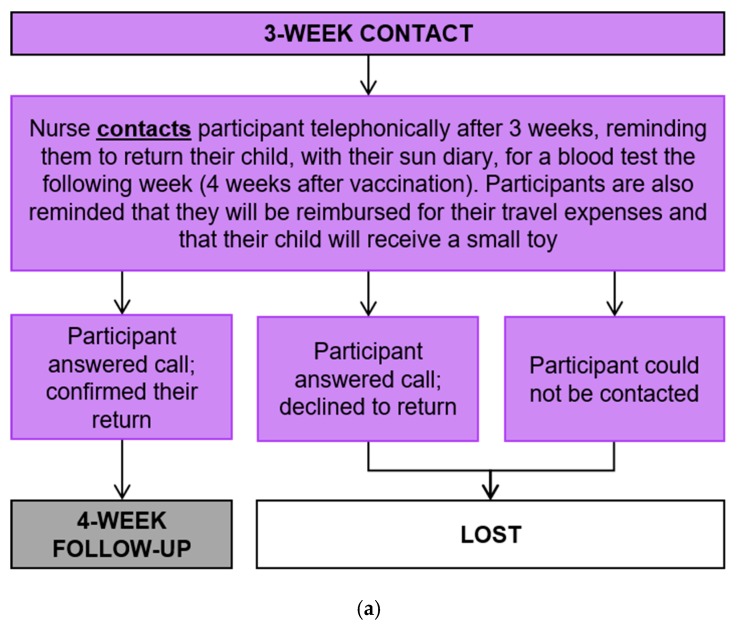

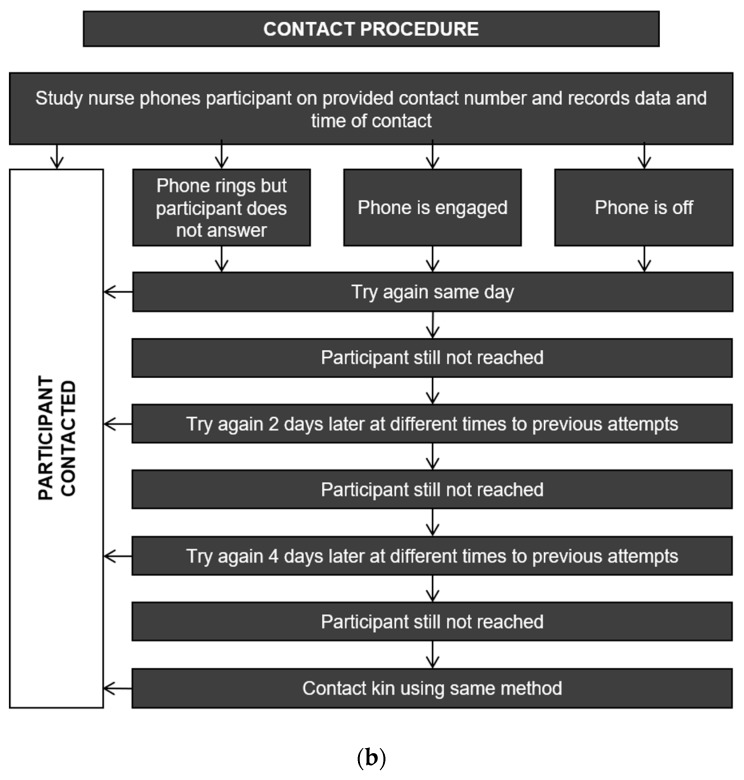
(**a**) Three-week contact process and (**b**) contact procedure.

Three weeks post-vaccination, the nurse contacted the mother, as per the predetermined contact procedure (Figure 6a,b) to organize their return for the blood sample to be taken and for them to return the completed sun diary. There were no data collected at this stage, however, each participant’s progression through this stage was captured on the participant flow checklist.

**Figure 7:**
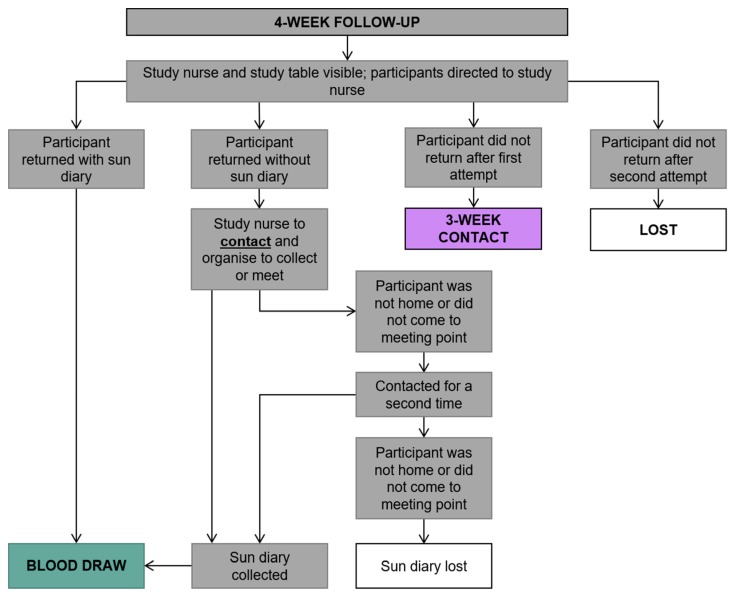
Four-week follow-up procedure.

**Figure 8:**
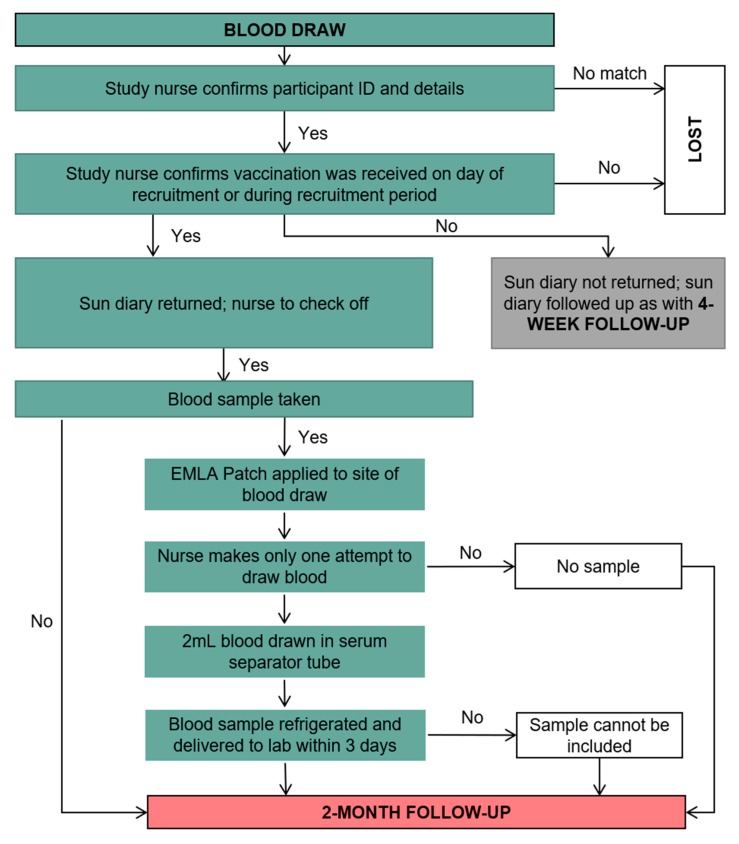
Blood sampling procedure.

**Figure 9:**
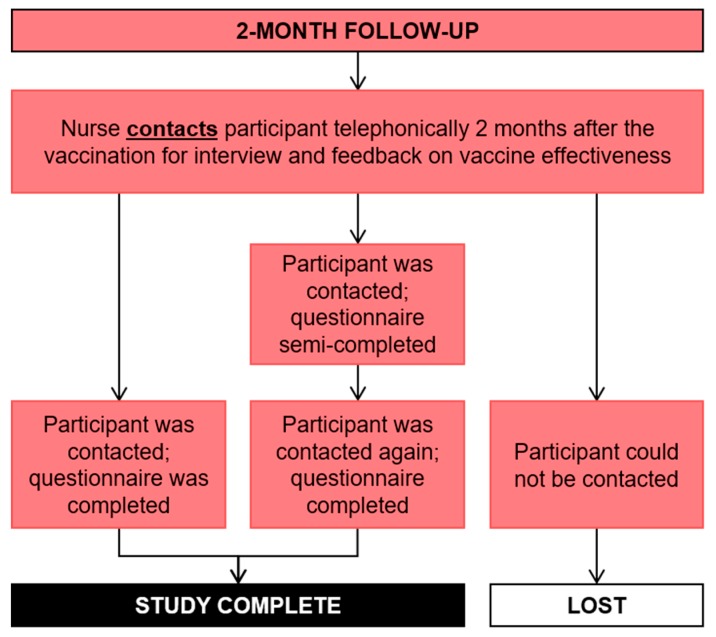
Two-month follow-up.

The following week, four weeks into the study (Figure 7), the participant returned to the clinic for the blood sample to be taken (Figure 8) and they were also asked to return the completed sun diary. Figure 7 also shows the procedure implemented to reduce loss to follow up. At this time, participants from the control clinic received the sun protective equipment. Two months post vaccination, the nurses contacted the participants to complete a second questionnaire; the same contact procedure was followed (Figure 9).

### 2.4. Tool Development

The data tool was created and designed with data entry and management tools in mind, such as Epidata [12], where data entry is easy but also can be regulated with checks to minimize data entry errors. Furthermore, the tool needed to create a database housing several tables that corresponded to the data capture sheets as well as capacity to upload environmental data and additionally, house a few tables specifically for the management aspect of the study. It was also envisaged that the tool would be able to track a participant’s movement through the study and send automated emails to the study team, prompting them of the next stages for a particular participant.

The tool was developed with Python [13] (Python Software Foundation) and the resulting database was created in SQLite [14] (SQLite Consortium). Both are easily accessible open source software with relatively easy coding languages to use. Python and SQLite were ideal choices because of the database requirement and data entry regulatory systems required. Another key advantage of using Python is that it can run without a web container, meaning that the website application is standalone and does not require a web server which requires more intensive setup.

The database included 10 tables, with the first table in the database being the participant’s table where the participant’s personal details, such as name, were stored with their corresponding assigned unique ID. After this point, no personal identifying information was stored. This was done so that when data were extracted no identifiable data were included. The remaining tables corresponded to the data capture sheets (enrollment checklist, questionnaire, Road to Health chart, nurse observations, sun diary and follow-up questionnaire), environmental data (site specific weather and UVR data) and management sheets (participant flow, flagged records).

By using a Python script, the date on which the child received their second measles vaccination in relation to the current date would be checked. It was planned that three weeks post-vaccination, the script would send an automated email to the study team notifying them of the elapsed time for a particular participant, along with all the required details (name, clinic and contact details) in order for the study team to contact and arrange the 4-week follow up visit. Once the script had sent a notification email for a participant, the script would then mark that participant as having been notified so multiple emails for the same participant were not sent. The same process was envisaged for the 2-month follow-up telephonic interview. This script was hosted on a personal computer and ran daily at 09:00. If the host computer was not running on a given day, the day was skipped. The script ran again when the host computer was next running at the predefined time ensuring that no participants were missed.

The structured database in SQLite linked all levels of data collected at the various stages for all participants. Each participant was assigned a unique identifier when they were enrolled in the study. This identifier was used in the database to maintain referential integrity (meaning the relationship between all the data in all the tables remains accurate to each individual).

The confidentiality of each participant was also maintained through this system. The database was created to allow only predetermined coded responses, for instance, 0 = yes, 1 = no, 777 = default, 888 = not applicable, 999 = missing. The default value was created automatically, thus allowing the researchers to see when a field has potentially not been captured rather than it being truly missing. Data for each variable were restricted to a data type, such as numeric, with predefined allowable values built into the database architecture. Date fields in all the forms were the same, where dates had to be entered in the correct format (dd/mm/yyyy). Some variables may have had further restrictions such as those based on previous responses or links to later sections (i.e., a stem and branch question approach) and these too were pre-programmed. The data capture forms in the tool were designed to exactly match the hard copy versions used in the field. The sun diary entry sheet used a check box system that exactly matched the hard copy version (see Supplementary Figure S1). When a check box was selected, it was coded with a 1; anything that was not selected was coded with a 0.

Code sheets were auto-generated for each data capture sheet. Each data entry sheet was created and tested individually before any data were entered. Testing involved ensuring strict limitations were put in place for data integrity.

Environmental and meteorological data, specifically temperature, relative humidity, and solar UVR levels, were collected throughout the study using specialized weather instruments at each study site. These data were exported from the equipment and imported into the database on a weekly basis when the researcher visited the site to download the data. Environmental and meteorological data were matched by their geographical location to one of the two clinic sites. This allowed all data to be stored in one location.

Another feature that was added to the tool was the ability to ‘flag’ records; this could be done at any point during data entry or later. When flagging a record, one could leave a comment, or details of why it was flagged. All flagged records were tabulated in a separate table which could be viewed. It was tabulated with the participant ID, the variable in question, the table that the variable came from, and a comment. This was extremely useful for double-checking queries during data entry, and after data entry if something seemed incongruous.

Data can be viewed at any point through the website application or in the SQLite database. The data can be easily exported from SQLite in .csv format for use in any other application such as statistical analysis software.

### 2.5. Tool Evaluation

One of the primary goals of any data management tool or data management practice is to ensure high quality data. Drawing on a published tool [15] we modified their data quality assessment framework to assess the functioning of our tool with respect to the clinical trial data housed in the tool’s database. Three criteria were applied in our assessment: completeness, conformance and plausibility. Completeness evaluated whether data were present and therefore, helped to identify the absence of data or missing data. Conformance is defined by whether data values adhered to pre-specified standards or formats. In this instance, value conformance was assessed to check whether the recorded data element agreed with constraint-driven data architecture defined in the rules dictionary. Plausibility assessed whether the values of the data points were believable when compared to an accepted range or distribution of values.

The nine data elements (i.e., demographics, baseline questionnaire, Road to Health chart, nurse weather observations, blood test results, sun dairy, environmental data, solar UVR data and the follow-up questionnaire) (see Table 1) were assessed using the three criteria and results are discussed below.

Each of these nine elements included the following sub-elements: 1. Demographics (obtained from the baseline questionnaire) comprised gender, age and population group; 2. The baseline questionnaire (see Supplementary File 1) which collected information on factors known to influence vaccine effectiveness as well as sun exposure; 3. Road to Health chart (data collected from the child’s Road to Health chart) which included date of birth, birth weight, birth length, birth head circumference, problems during pregnancy/birth/neonatally, APGAR (Appearance, Pulse, Grimace, Activity, and Respiration) score at 1 min and 5 min, gestational age, if they received other immunizations prior to the first measles vaccination (e.g., Polio/Hepatitis), if they received the first measles vaccination, date of the first measles vaccination and batch number of the first measles vaccination; 4. Nurse weather observations included date, time, clinic, today’s weather (sunny, some cloud cover, completely overcast, raining), temperature feel, people waiting outside the clinic, is there shade, are they in the shade, number of people in the shade, number of participants in the shade, people waiting in the sun, people waiting inside; 5. Blood test: measles blood titre results received from the laboratory; 6. Sun diary, we asked that the following 14 variables (were they inside, were they in a car/bus/etc., were they in the sun, were they in the shade, were they wearing a hat, sunscreen, sunglasses, were they wearing a dress, short sleeves, long sleeves, shorts, trousers or swimming costume) were recorded three times a day for a week (see Supplementary Figure S1); 7. Environmental data recorded on the weather monitors included maximum temperature, minimum temperature, average temperature, humidity, dew point, wind speed, highest wind speed, wind chill, heat index, THW (Temperature, Humidity, Wind) index and rainfall; 8. Solar UVR data; and 9. The follow-up questionnaire included if the mother liked the sun protection equipment, was it easy to use, if the child liked using it and any further comments.

## 3. Results and Discussion

### 3.1. Study Sample

**Figure 10:**
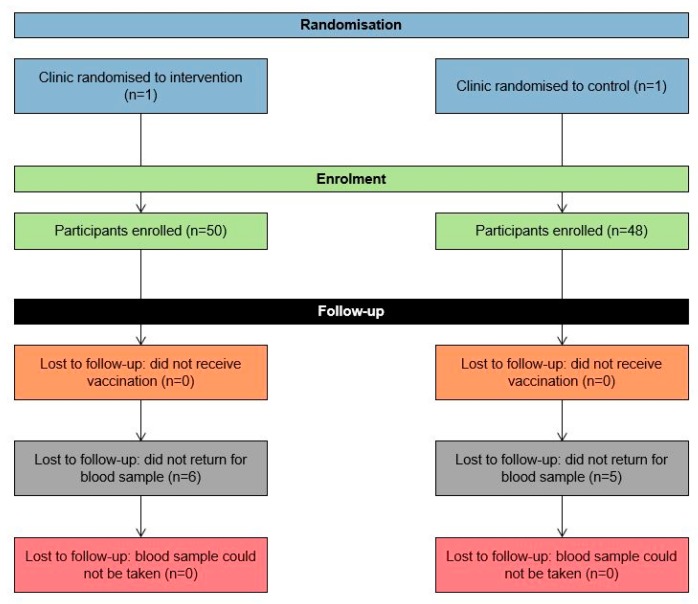
CONSORT flow diagram providing details on the enrolment, allocation and follow-up frequencies for the study.

A total of 98 children from two clinics (intervention group: n = 50; control group: n = 48) and all from the Black African population group participated in the study (Figure 10) with recruitment taking place from December 2015 to March 2016. Eleven children did not attend the follow-up visit for the blood sample test (six in the intervention group and five in the control group), thus, a total of 87 (89%; 95% CI 81%–94%) children had blood drawn for antibody level testing approximately four weeks post-vaccination.

### 3.2. Evaluation of the Data Management Tool

The data entry tool and the database bringing together all datasets and components of the study fulfilled the main criteria for clinical data management: data integrity [4]. Table 1 provides the results of the data entry tool assessment using the adapted framework of three criteria: completeness, value conformance and plausibility.

For the data element category of demographics, completeness, value conformance and plausibility were high due to the legal values in the database architecture and cross-checking with date of birth on the Road to Health card. Twenty-one elements from the baseline questionnaire were assessed (see Supplementary File 1 for the corresponding Question numbers). Six values in the questionnaire were default-coded ‘777’ and were corrected as ‘missing’. For Q11, only weight was included in the assessment as height was poorly completed by the respondents.

For the Road to Health chart data, implausibility was noted among some of the date of birth records and records marked ‘unknown’ were amended to ‘missing’ data. For Q7 on the chart, the data was missing on the card completed by primary healthcare nurses.

Nurse observations of the weather conditions each day had some incomplete or incomprehensible records, especially pertaining to date, where in a few (five) records, the year in the date was incorrectly recorded, however, these mistakes were easy to amend based on the dates of the study period. Several days were not completed by the Nurses, hence missing data and lack of completeness.

The diary used a checkbox system for record-keeping (See Supplementary Figure S1), hence, completeness was 100%. However, further investigation revealed that only 296 (17%) records had a positive selection indicating the extent of how poorly the diaries were completed. This was likely to due to losing interest (study fatigue). Several other studies that have used self-completed diaries had similar challenges [16]—this can be overcome with frequent prompting from field workers and through the use of electronic diaries [16,17]. In the dairies, several dates were missing; however, these could, in many cases be rectified in post-processing if the starting date was recorded.

For the environmental data, there were no missing data, the instruments operated without error during the entire project period and data were easily uploaded to the tool rather than entered manually.

The solar UVR data were not uploaded to the tool because of equipment malfunctions and data integrity challenges which required amendments prior to processing. There were problems with instrument failure and some of the solar UVR instruments stopped recording or recorded erroneous data. Future studies should use more reliable solar UVR measurement instruments to avoid data loss of this nature.

The follow-up questionnaire was well implemented and complete, and all data conformed to the required values and were plausible.

The data entry tool made data entry easy and fast, and reduced possible data entry mistakes using legal values, stem and branch questions using an auto-completed default value and check boxes. No data were lost, and when queries arose, they were quickly resolved. Furthermore, the tool included a home table which showed all the entered participants’ identifier codes, and what stages had been completed for each participant and what stages were still incomplete. This allowed the researcher to quickly check what was outstanding, and therefore, manage all the incoming data. The tool allowed referential integrity to be maintained through all the data capture forms by using a unique identifier. It further ensured confidentiality of participants to be maintained by ensuring that personal details were stored in one table only (password protected) and when exporting the data for analysis, that table was excluded.

### 3.3. Limitations

During the study preparation stage, it was planned that the nurses would have access to laptops, the Internet and emails in order to enter the data as it was generated; however, due to the remoteness of the sites and for safety reasons, it was decided that this would not be feasible. There were also no facilities for the blood samples to be transported daily, so they needed to be done in batches, collected weekly and transported to the laboratory. As a result, the study team needed to cluster participants’ blood sampling days so that the samples were collected on one day per week and collected that same day. The data entry was done in weekly batches when the study team visited the sites to collect the data and blood samples. This meant that the automated emailing for participant contacting and managing their progression through the study was handled manually by the study primary investigator. Our follow-up rates were high as a result of multiple follow-up calls made to the participants by the research nurses.

One of the aims of this study was to assess recruitment, consent and follow-up. Unfortunately, the protocol for collecting information on recruitment rates was not adequately implemented, despite the implementation of the tailored clinical trial data management tool, so this information was not available. Anecdotally, almost all mothers who were asked to participate in the study consented; however, in general, the number of clinic attendees presenting at the clinic was lower than anticipated. Our original estimates on number of clinic attendees were based on estimations from the nurses at the rural clinics, as well as on provincial-level records for rural clinics that may have varied greatly between clinics located within a province. As a result of low presentation for vaccination, our anticipated recruitment period of three months was extended to four months in order to meet our sample size requirements.

An issue that emerged throughout the data was the date fields. They were set up to allow only dates which had to be entered in the correct format (dd/mm/yyyy), but there was still room for error, for instance, 5 February 2016 could accidentally be entered as 5 February 2015. However, this was easy to correct in post-processing by comparing the date of birth multiple times from different sources but also asking the participant’s age in years and months, as we did.

A shortfall of the tool was the inability to accommodate the solar UVR data, although this was not a poor reflection of the tool, but rather due to poor solar UVR data quality due to instrument failure. The meteorological data were easily incorporated into the tool, suggesting that had more consistent solar UVR data been collected, it would also have been included in the tool with ease.

The development of the tailor-made clinical data management tool applied in this environmental health study was labor-intensive and required skills in programming and information technology. Other clinical data management systems have similar challenges; for example, they require sophisticated information technology to function [18]. Despite these challenges, our tool proved to be adequate in managing a relatively large dataset comprising clinical, environmental and behavioral data for analysis and interpretation. Future environmental health randomized clinical trials may consider modifying the tool used here or investigating other software, such as REDCap (Research Electronic Data Capture) [19], which may be able to incorporate environmental and meteorological data.

## 4. Conclusions

Our tool provides an efficient data capture and management solution. In this study, we customized everything to meet the needs of the study. Using this tool as a framework, one could customize it to meet the needs of most studies of this nature; however, this would require some knowledge of Python and SQLite. Beyond the database and management benefits of the tool, the data entry checks, ability to flag records and the email notifications were extremely valuable, highlighting the benefits and versatility of creating bespoke tools such as this one.

At a time when the relations between environmental exposures and human health are under the spotlight, especially air pollution and climate change [20], there is a need for environmental health research of the highest quality underpinned by high-quality data. The development of a tailor-made clinical trial data management system for an environmental health intervention study was shown to be possible. Implementation of the tool was relatively easy and helped to bring together multidisciplinary datasets. Using the tool to track a participants’ stage in the project and ensuring that close contact was maintained between the researchers, the research nurses and the participants ensured high quality data. Further research and development should be done to improve on such tools, for example, by adding standard operating procedures to track recruitment rates.

## Figures and Tables

**Table 1:** Tool assessment framework adapted from Lee et al. [15] with total data elements by category and number of data elements adhering to the concept criteria.

Data Element Category	*n* Elements Assessed	*n* in Dataset	Number/% of Data Elements Adhering to the Concept Criteria		
			Completeness	Value Conformance	Plausibility
Demographics	3	98	Gender (98) Age (95) Pop group (98) Total 291/294 * 100 = 99%	291 (100%)	291 (100%)
Baseline questionnaire	21	98	(Q6) 97; (Q7) 98; (Q8) 98; (Q9) 95; (Q10) 98; Q(11) 94; (Q12) 92; (Q13) 98; (Q15) 98; (Q16) 98; (Q17) 98; (Q18) 98; (Q19) 98; (Q20) 98; (Q24) 97; (Q25) 97; (Q26) 97; (Q30) 95; (Q31) 93; (Q32) 96; (Q36) 97; Total: 2030/2058 * 100 = 98%	2052 (99%)	2058 (100%)
Road to Health chart	12	98	(Q1) 98; (Q2) 96; (Q3) 97; (Q4) 97; (Q5) 97; (Q6 1min) 93; (Q6 5min) ^#^ 93; (Q7) 15; (Q8) 98; (Q9a) 98; (Q9b) 98; (Q9c) 98; Total: 1079/1176 * 100 = 92%	100%	1167/1176 * 100 = 99%
Nurse weather observations	12	82 days in each clinic, total 164 ^+^	(Date) 142; (Time) 0; (Q1) 142; (Q2) 142; (Q3) 142; (Q4) 142; (Q5 141; (Q6) 141; (Q7) 141; (Q8) 141; (Q9) 141; (Q10) 140 Total: 1555/1968 * 100 = 79%	1958 (99%)	1956 (100%)
Blood test	1	88	87 (99%)	87 (100%)	87 (100%)
Sun Diary	14 variables recorded 3 times a day for 7 days = 1 827 records each with 14 variables = 25 578	87	(Time) 1827 (Date) 1296; 1827; 1827; 1827; 1827; 1827; 1827; 1827; 1827; 1827; 1827; 827; 1827; Total: 25,047/25,578 * 100 = 98%	100%	N/A
Environmental data	11 x 2 (for each clinic)	125 (days)	100%	100%	100%
Solar UVR data (total days)	4 and then 6 instruments^@^	125 (days) 28 (4) 97 (6)	141/(112 + 582) * 100 = 20%	N/A	N/A
Follow-up questionnaire	6	88	(Q1) 86; (Q2) 84; (Q3) 83; (Comments) 87 Total: 340/352 * 100= 96%	100%	100%

Note. * Total number of items recorded for this element category.^#^ (Q6 1 min) and (Q5 1 min) refer to the APGAR (Appearance, Pulse, Grimace, Activity, and Respiration) Score recorded at 1 min and 5 min. @ 4 and then 6 instruments as two additional instruments were added when it became apparent the equipment was not functioning optimally. + Excluded weekends and public holidays.

## Data Availability

The datasets analyzed during the current study are not publicly available due to the data forming part of a doctoral thesis which is still to be submitted but are available from the corresponding author on reasonable request.

## Supplementary Materials

The following are available online at https://www.mdpi.com/1660-4601/17/2/402/s1, Figure S1: Screenshot of the dairy entry form in the tool. File S1: Full Study Questionnaire. The source code for the tool can be accessed at https://gitlab.com/pnalbers/askr.
