# Supplementary material for: Clinical Trial Data Management in Environmental Health Tailored for an African Setting
Source: Int J Environ Res Public Health. 2020 Jan 8;17(2):402. doi: 10.3390/ijerph17020402 (PMC7013767; doi:10.3390/ijerph17020402)
Supplement: Supplementary file 1 [file ijerph-17-00402-s001.zip › Questionnaire.pdf]

QUETIONNAIRE
PRIVATE AND CONFIDENTIAL

Unique identifier code: ☐ ☐ ☐ ☐

Title of research project: Environmental effects on vaccines given to children

Research ethics clearance numbers: EC013-4/2015 (Medical Research Council Research Ethics Committee)

Permissions: Proposal: 4/2/2, Limpopo Department of Health (Contact: Shamila Latif)

Researcher contact information: Dr Caradee Wright (Telephone 012 339 8543 or 082 677 4037)

Participant information

Please read the information sheet and sign the consent form (two copies – you may keep one signed copy) if you agree to participate before answering the questions below.

QUESTIONS

Please answer all of the questions below by placing a cross (x) in the box of your choice of answer.

GENERAL QUESTIONS ABOUT YOU AND YOUR CHILD

1. Are you this child's:

   a. Mother ☐ (1.1)

   b. Guardian ☐ (1.2)

   c. Family relation ☐ (1.3)
   (i.e. sister, aunt, grandmother)

2. Is your child:

   a. Black ☐ (2.1)

   b. Indian/Asian ☐ (2.2)

   c. White ☐ (2.3)

   d. Coloured ☐ (2.4)

   e. Other: please write it here____(2.5)

3. What age are you today?

   a. 18 - 25 years ☐ (3.1)

   b. 26 - 35 years ☐ (3.2)

   c. 36 - 45 years ☐ (3.3)

   d. 46 - 60 years ☐ (3.4)

   e. Older than 61 years ☐ (3.5)

4. How old is your child who is here for the measles booster vaccination today?

   a. Years______________ (4.1) Months______________ ☐ (4.2)

5. Is your child:

   a. Male ☐ (5.1)

   b. Female ☐ (5.2)

6. Does your child have oculocutaneous albinism (this means that your child has white skin instead of black skin)?

   a. No ☐ (6.1)

   b. Yes ☐ (6.2)

7. What is the HIV status of your child?

   a. HIV status of your child: Positive ☐ (7.1) Negative ☐ (7.2)

8. What colour is your child's skin (see the colour chart at the back of this questionnaire)?

   a. Very fair ☐ (8.1)

   b. White ☐ (8.2)

   c. Light brown ☐ (8.3)

   d. Brown ☐ (8.3)

   e. Dark brown ☐ (8.4)

   f. Very dark brown / Black ☐ (8.5)

9. Does your child get sunburnt in the sun? Sunburn means when their skin changes colour, gets sore or starts to blister after being in the sun.

   a. No ☐ (9.1)

   b. Yes ☐ (9.2)

10. How sensitive do you think your <u>child's skin</u> is to the sun? Sensitive means that they get sunburned quickly when they are in the sun

    a. Very sensitive ☐ (10.1)

    b. Sensitive ☐ (10.2)

    c. Moderately sensitive ☐ (10.3)

    d. Not sensitive at all ☐ (10.4)

11. What is the weight and height of your child today (you can ask your nurse)?
    a. Weight__________________kg (11.1)
    b. Height__________________ cm (11.2)
12. Is your child on any medication today? Medication refers to medicine given to you by the doctor / clinic nurse or medicine that you buy from a pharmacy or medicine you get from your traditional doctor.
    a. No ☐ (12.1)
    b. Yes ☐ (12.2) if yes, please write the name of the medication(s) and how often the child must take the medicine here:

**Name of medication**	**How many times a day is the medicine taken?**

13. What is the child's usual level of physical activity, such as running, walking, playing?
    a. Very active (means lots of running, playing etc) ☐ (13.1)
    b. Moderately active ☐ (13.2)
    c. Not very active ☐ (13.3)
14. Does the mother, father or usual caregiver/guardian of the child smoke?
    a. No ☐ (14.1)
    b. Yes ☐ (14.2)
15. Does anyone smoke inside the house of this child?
    a. No ☐ (15.1)
    b. Yes ☐ (15.2)

16. Was this child breastfed at the time he/she had the <u>first</u> Measles vaccination?

a. No ☐ (16.1)

b. Yes ☐ (16.2)

c. Don’t know ☐ (16.3)

17. Is this child still being breastfed now?

a. No ☐ (17.1)

b. Yes ☐ (17.2)

c. Don’t know ☐ (17.3)

18. Did <u>this child’s mother</u> have the Measles vaccinations when she was a baby?

a. No ☐ (18.1)

b. Yes ☐ (18.2)

c. Don’t know ☐ (18.3)

19. Has <u>this child’s mother</u> had measles?

a. No ☐ (19.1)

b. Yes ☐ (19.2)

c. Don’t know ☐ (19.3)

20. How do you feel about children playing in the sunshine?

a. It is healthy ☐ (20.1)

b. It is harmful ☐ (20.2)

c. Not sure / Don’t know ☐ (20.3)

TRAVEL TO CLINIC

21. When you take your child to the clinic, how do you usually travel?

a. Bus ☐ (21.1)

b. Taxi ☐ (21.2)

c. Train ☐ (21.3)

d. Walk ☐ (21.4)

e. Private car ☐ (21.5)

f. Other: please write it here__________________________________(21.6)

22. How long does it usually take you to travel to the clinic?

a. Less than 10 minutes ☐ (22.1)

b. More than 10 but less than 30 minutes ☐ (22.2)

c. Between 30 minutes and one hour ☐ (22.3)

d. More than an hour ☐ (22.4)

23. At the clinic, is there sometimes a long queue or line in which you have to wait?

a. No ☐ (23.1)

b. Yes ☐ (23.2)

c. Don't know ☐ (23.3)

24. When you arrive at the clinic, where do you usually wait?

a. Shaded area ☐ (24.1)

b. Unshaded area ☐ (24.2)

c. Inside ☐ (24.3)

25. How long do you usually wait for in the queue/line?

a. Less than 15 minutes ☐ (25.1)

b. 15 – 30 minutes ☐ (25.2)

c. 30 minutes – 1 hour ☐ (25.3)

d. More than 1 hour ☐ (25.4)

WAITING AT CLINIC

<u>If you usually wait **inside** the clinic, please skip to Question 30.</u>

26. If you do <u>wait outside</u> at the clinic, do you use sun protection for your child (by sun protection, we mean a hat, an umbrella, a sunscreen lotion or other means of protecting yourself from the sun)?

a. No ☐ (26.1)

b. Yes ☐ (26.2)

c. I wait with my child inside ☐ (26.3)

27. If you do use sun protection on your child when you wait outside at the clinic, how often do you use it?

a. Usually ☐ (27.1)

b. Sometimes ☐ (27.2)

c. Seldom ☐ (27.3)

d. Never ☐ (27.4)

e. I wait with my child inside ☐ (27.5)

28. If you do use sun protection on your child when you wait outside at the clinic, what sun protection do you use?

a. Hat or cap ☐ (28.1)

b. Sunscreen (cream or lotion to protect skin) ☐ (28.2)

c. Long-sleeved shirt ☐ (28.3)

d. Long pants or trousers ☐ (28.4)

e. Umbrella ☐ (28.5)

29. If you do apply sunscreen to your child when you wait outside at the clinic, where do you usually apply it to on their body?

a. Face ☐ (29.1)

b. Arms ☐ (29.2)

c. Legs ☐ (29.3)

d. Hands ☐ (29.4)

e. Back and shoulders ☐ (29.5)

f. Did not apply sunscreen to any body parts ☐ (29.6)

CHILD'S TIME SPENT OUTSIDE

30. Where does your child usually spend their time on weekdays?

   a. Mostly inside ☐ (30.1)

   b. Mostly outside ☐ (30.2)

31. Where does your child spend most of their time on weekend days?

   a. Mostly inside ☐ (31.1)

   b. Mostly outside ☐ (31.2)

32. If your child did spend time outdoors during daylight hours in the past week, was your child mostly in the shade or mostly out in the open in the sunshine?

   a. Shade ☐ (32.1)

   b. Open/sun ☐ (32.2)

33. If your child did spend time outdoors during daylight hours in the past week, about how many hours does your child usually spend in the sunshine each day?

   a. Less than 1 hour ☐ (33.1)

   b. 1 hour ☐ (33.2)

   c. 2 hours ☐ (33.3)

   d. 3 hours ☐ (33.4)

   e. More than 3 hours ☐ (33.5)

34. If your child did spend time outdoors during daylight hours in the past week, did your child usually use the following to protect their body from the sun? (mark as many as apply)

a. Hat or cap ☐ (34.1)

b. Sunscreen (cream or lotion to protect skin) ☐ (34.2)

c. Long-sleeved shirt ☐ (34.3)

d. Long pants or trousers ☐ (34.4)

e. Umbrella ☐ (34.5)

35. If you did apply sunscreen to your child's body, which parts of the body was the sunscreen lotion applied?

a. Face ☐ (35.1)

b. Arms ☐ (35.2)

c. Legs ☐ (35.3)

d. Hands ☐ (35.4)

e. Back and shoulders ☐ (35.5)

f. Did not apply sunscreen to any body parts ☐ (35.6)

36. If your child did spend time outdoors during daylight hours in the past week, did your child ever get sunburnt (when their skin changed colour, went red or purple or got blisters)?

a. No ☐ (36.1)

b. Yes ☐ (36.2)

Thank you for completing the questionnaire. Please hand it back to the Study Nurse.
