# Supplementary figures and images for: Clinical Trial Data Management in Environmental Health Tailored for an African Setting
Source: Int J Environ Res Public Health. 2020 Jan 8;17(2):402. doi: 10.3390/ijerph17020402 (PMC7013767; doi:10.3390/ijerph17020402)

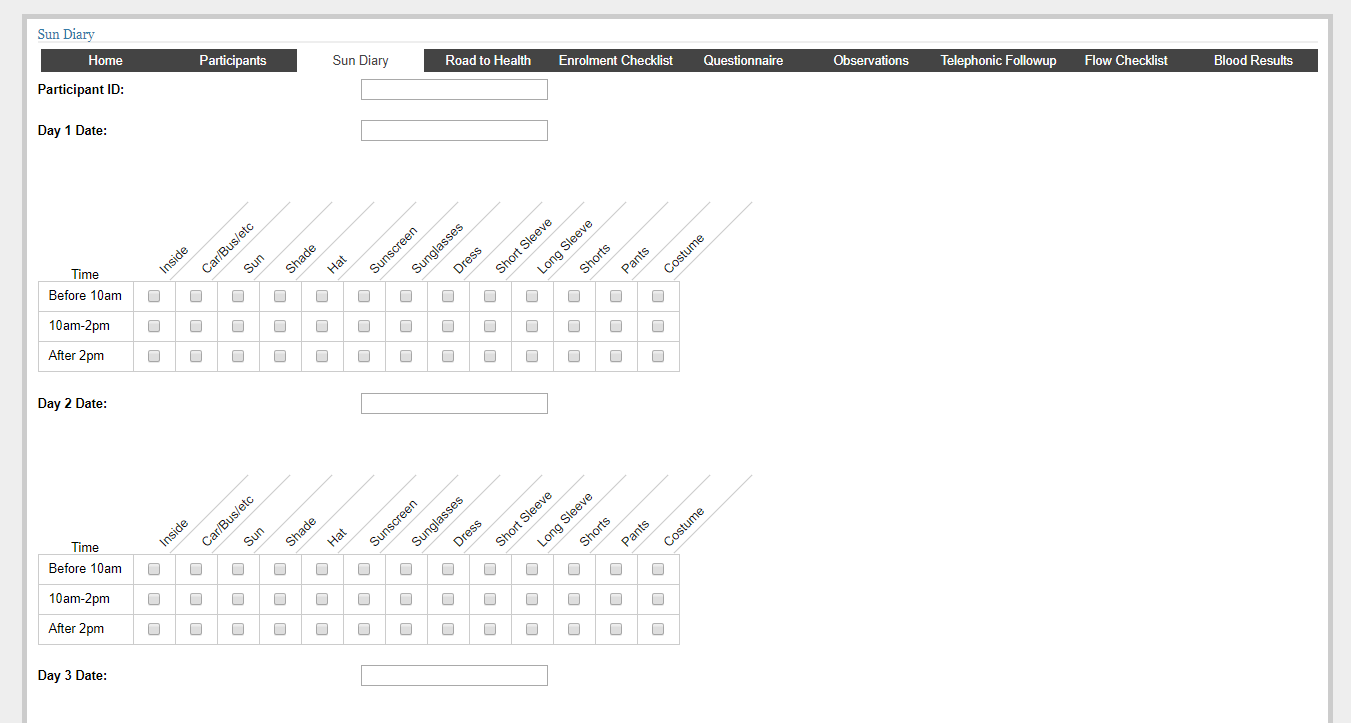

Supplement: Supplementary file 1 [file ijerph-17-00402-s001.zip › Sundiary entry sheet.png]
